# Dyspeptic Letters

**Published:** 1860-07

**Authors:** 


### DYSPEPTIC LETTERS.

NEW-YORK, Feb. 16th, 1860.

DEAR SIR: You have a most tremendous sight to learn, if you would get along swimmingly in this mundane sphere. As far as health and medicine are concerned, you will have to begin at forty degrees below A B C. You have dyspepsia and liver complaint; these give debility and promote discharges which reäct and increase the debility. Some pills act on the stomach, such as Ipecac or Tartar Emetic. Some act on the upper portion of the bowels, as Rhubarb. Some act on the rectum, or lower bowel, as Aloes; others act on the whole, by acting first on the liver: and yet you say my pill acted as any pill would. I gave you a pill to act on the liver, and the difference in color of the action of such a pill and one which merely acts on the bowels, is as different as black or green from yellow or red.

You say you don't think you need medicine. If you know so much, you ought to have cured yourself. If you consult a physician, you ought to make up your mind to take his advice and his medicine too; he don't want to know what you think, because he knows your "thinks" are not worth a button.

You are young—you have a good constitution, and have no serious disease at this time; yet what you complain of will in time destroy both body and mind. Already you complain that your mind is not clear, and that when you go to bed, if you can not go to sleep at once, you lie and think, and sweat, and fret, and fidget, and toss, and tumble for hours together. You complain also of an emptiness before meals, and a distressing palpitation of the heart, which incapacitates you from exercise. These symptoms are present in a greater or less degree in all cases of torpid liver and a weak digestion. Some hearts palpitate so much on going to bed as to prevent sleep for hours; it or some of the larger blood-vessels beat and tick and throb and thump and click and breeze for half a night.

Some minds in dyspepsia are not only not clear, but are incapacitated for connected thought. The attention can not be fixed steadily on any thing, and horrible fears come on; dread of loss of character; of loss of reason; of loss of friends; of loss of money; at other times most terrible fancies run riot through the brain; temptations of darkest import assail the heart, and nothing is safe, nobody is safe—it is a perfect "mania," not a "Potu," but "mania Phagi," not a madness from drinking, but a madness from eating, and from this, some of the best and most useful and successful men go down to the terrible grave of a suicide every year, every month. A drunkard seldom kills himself, a dyspeptic often does; and the coroner's verdict is, "Died by his own hand while in a state of mental aberration." If they do not murder themselves, they live only to be the pest and plagues of themselves and those who are nearest and dearest to them.

One of the reasons of the incurability of dyspeptics is, that they have not sufficient force of will, sufficient persistence of purpose, to follow out the continuous plan necessary to their restoration. To-day they think they would be willing to do any thing, to-morrow they determine they will do nothing. At one time they will swig cod-liver oil by the gallon, rub goose-grease on their nose, make ducks of themselves at water-cures, drink their own urine by the quart; all these things are done, as physicians know, constantly. At another time they will assert, like you, that they don't think they need medicine; that they are tired to death of medicine, and had rather die than take another dose. A great name told us once, having written for advice, that he would take no medicine from any man, especially if he did not know what it was, and gave his reason for it. Now a fool's reasons always confirm him in his foolishness. It was, that he had been taking medicine of his own prescribing for twenty years, and it had done him no good. We wrote to him that he could go to grass. We don't like to be made a fool of.

Years rolled on, and he rolled with them into the Niagara River. From these things I would be glad to rescue you, but then you must not bother me with your "thinks" and suggestions. Tell me what your actual sensations are; then I will tell you how to remove them, and send or describe the instrumentalities. I have no objections to tell persons what I give, or why I give it—I always prefer it when I am satisfied that the patient has any sense; if he has not, what's the use of wasting time?

Hoping that you may be profited by these hints, I send in another paper such directions as I think are applicable to your case. Duly yours.

A physician must be crusty sometimes. The above was written for a young gentleman who subsequently became a very obedient patient, and we have published it, for the suggestions contained may be practically useful to a large class of persons; the patient was laboring under dyspeptic debility, constipation, etc.

